# Effect of Statin Therapy on Clinical Outcomes in Patients With Cardiovascular Risks: A Systematic Review and Meta-Analysis

**DOI:** 10.7759/cureus.88238

**Published:** 2025-07-18

**Authors:** Kiyan Ghani Khan, Priyadeep Kaur, Mehak Bhagat, Huda K Klair, Maryam Bakhtawar, Jorge Manuel Aldea Saldaña, Hasiya M Bello, Hussein Attia Hussein Mahmoud, Mahesh Babu, Tanvi Mahajan, Usman Khan, Manju Rai

**Affiliations:** 1 Internal Medicine, Baqai Medical University, Karachi, PAK; 2 Internal Medicine, Punjab Institute of Medical Sciences, Jalandhar, IND; 3 Internal Medicine, Government Medical College, Amritsar, Amritsar, IND; 4 Internal Medicine, Fatima Memorial Hospital (FMH) College of Medicine and Dentistry, Lahore, PAK; 5 Internal Medicine, Beihua Medical University, Jilin City, CHN; 6 Cardiology, Universidad Peruana de Ciencias Aplicadas, Lima, PER; 7 Emergency Medicine, Buraydah Central Hospital, Buraydah, SAU; 8 Diagnostic Radiology, Heliopolis Hospital, Cairo, EGY; 9 Internal Medicine, Dr. PSI Medical College, Andhra Pradesh, IND; 10 Internal Medicine, Maharishi Markandeshwar Medical College and Hospital, Dinanagar, IND; 11 General Practice, Akhtar Saeed Medical and Dental College, Lahore, PAK; 12 Biotechnology, Shri Venkateshwara University, Gajraula, IND

**Keywords:** all-cause mortality, cardiovascular disease, cardiovascular hospitalization, ldl cholesterol, mace, statin therapy

## Abstract

Statin therapy effectively reduces low-density lipoprotein (LDL) cholesterol, thereby lowering the risk of atherosclerosis and cardiovascular (CV) events. This systematic review and meta-analysis assessed its impact on all-cause mortality, CV mortality, major adverse cardiovascular events (MACE), such as heart attack, stroke, coronary revascularization, and CV hospitalization in adults with CV risk factors or established cardiovascular disease (CVD). A systematic search of PubMed, Google Scholar, and Cochrane Central (2013-2024) included randomized controlled trials, prospective cohorts, and retrospective studies. Primary outcomes were all-cause mortality, CV mortality, MACE, and CV hospitalization. A random-effects model was employed, with heterogeneity assessed using the I² statistic. Seven studies, comprising 506,813 patients (118,491 statin users and 388,322 non-users), with a mean follow-up of 3.7 years, were included. Statin therapy significantly reduced all-cause mortality (relative risk (RR) 0.60, 95% confidence interval (CI): 0.43-0.83, p<0.00001, I²=91%), and MACE (RR 0.79, 95% CI: 0.71-0.87, p<0.00001, I²=0%). The high heterogeneity observed in mortality outcomes likely reflects differences in study populations, statin types, and baseline risk profiles. A non-significant trend was observed toward CV mortality (RR 0.74, 95% CI: 0.53-1.02, p<0.00001, I²=90%). Additionally, no significant reduction in CV hospitalizations was observed (RR 0.97, 95% CI: 0.83-1.13, p=0.58, I²=0%). Statin therapy significantly lowers all-cause mortality and MACE, reinforcing its role in CV risk management. However, its effect on CV mortality and CV hospitalization remains uncertain, warranting further investigation into complementary strategies for reducing hospital admissions.

## Introduction and background

Cardiovascular diseases (CVDs) represent a significant global health challenge, accounting for an estimated 17.9 million deaths annually [1]. These conditions, which include cerebrovascular disease, coronary heart disease, and peripheral artery disease, affect both developed and developing nations. Atherosclerosis, the buildup of plaque inside the arteries, is the common underlying mechanism for most of these conditions. While advancements in medical treatment and preventive measures have contributed to a decrease in CVD-related mortality in developed countries, the burden remains substantial in developing regions due to limited healthcare access and prevalent risk factors [2].

CVD risk factors can be classified into modifiable factors, such as obesity, diabetes, hypertension, and hypercholesterolemia, and non-modifiable factors like age, gender, and genetic predisposition [3]. The ongoing process of urbanization and lifestyle changes has contributed to the rising rates of CVD, underscoring the need for effective interventions in both primary prevention (preventing disease in high-risk individuals) and secondary prevention (preventing recurrence in patients with established disease).

Statin therapy has emerged as a cornerstone in the management of CVDs, primarily by lowering low-density lipoprotein (LDL) cholesterol. Statins inhibit 3-hydroxy-3-methylglutaryl-coenzyme A (HMG-CoA) reductase, the enzyme responsible for cholesterol synthesis in the liver, thereby reducing circulating cholesterol levels and slowing the progression of atherosclerosis [4,5]. Statins are widely used in both primary and secondary prevention, with various formulations, such as atorvastatin and rosuvastatin, offering differing potencies [6]. Despite their benefits, statin use may be limited by side effects like muscle soreness, liver enzyme elevation, and increased risk of diabetes [7]. However, with proper monitoring and education, patient adherence can be optimized.

This systematic review and meta-analysis specifically focuses on adults with cardiovascular (CV) risk factors or established CVDs, including heart failure and coronary artery disease. It aims to evaluate the impact of statin therapy on four clinical outcomes: all-cause mortality, CV mortality, major adverse cardiovascular events (MACE, a composite of CV death, heart attacks, and strokes), and CV hospitalization. Previous meta-analyses have shown consistent benefits of statins in reducing mortality and MACE, but conflicting findings regarding their impact on CV hospitalization. This lack of clarity highlights the need for further evidence, particularly in diverse patient populations and across various study designs.

## Review

Methods

We adhered to the 2009 PRISMA (Preferred Reporting Items for Systematic Reviews and Meta-Analyses) guidelines for reporting in this study [8]. Given the nature of the study as a meta-analysis, Institutional Review Board (IRB) approval and patient informed consent were not required.

Search Strategy

The study conducted a comprehensive search across three databases: PubMed, Google Scholar, and the Cochrane Central Registry of Controlled Trials. The search period was limited to 2013-2024 to ensure the inclusion of recent evidence reflective of contemporary clinical practices, statin formulations, and diagnostic criteria. Earlier studies were excluded to minimize variability resulting from outdated management guidelines or statin dosing practices. The search utilized the following keywords: 'heart failure,' 'HF,' 'left ventricular dysfunction,' 'heart failure with preserved ejection fraction' (HFpEF), 'heart failure with reduced ejection fraction' (HFrEF), and 'heart failure with mid-range ejection fraction' (HFmrEF). These were combined with terms such as 'statin,' 'statins,' 'lipid-lowering therapy,' 'dyslipidemia therapy,' and specific statins like 'simvastatin,' 'atorvastatin,' 'rosuvastatin,' 'pitavastatin,' 'pravastatin,' and 'lovastatin.' Additionally, keywords including 'all-cause mortality,' 'cardiovascular mortality,' 'hospitalizations,' as well as 'lipid,' 'lipids,' 'cholesterol,' 'lipoprotein,' and 'lipoproteins,' were used to ensure comprehensive identification of relevant studies. Additional searches for potential trials involved reviewing the references of relevant review articles, as well as abstracts from meetings of the European Society of Cardiology (ESC), American Heart Association (AHA), American College of Cardiology (ACC), European Society of Atherosclerosis (EAS), and the National Lipid Association (NLA).

Heart failure and its subtypes (HFpEF, HFrEF, HFmrEF) were included in the search strategy because many patients with CV risk or established CVD fall within this population, and prior studies have shown divergent outcomes with statin use in heart failure cohorts. Including these terms ensured comprehensive retrieval of relevant studies across the broader CV risk spectrum.

Study Selection

The inclusion criteria encompassed randomized controlled trials (RCTs), prospective cohort studies, and retrospective studies involving adults with CV risk factors or diseases. To evaluate the efficacy of statin therapy, these studies included a comparison group receiving either a placebo, non-statin therapy, or no treatment. Only studies that focused on key outcomes such as all-cause mortality, CV mortality, MACE, or CV hospitalization were selected. Additional inclusion criteria included a follow-up period of at least 12 months, CV events as either the primary or secondary outcomes, the presence of a control group, a minimum of 50 participants, and patients aged 18 years or older. Additionally, only research published in English was considered.

On the other hand, studies were excluded if they were case reports, case series, reviews, letters, or non-peer-reviewed articles. Populations primarily consisting of pediatric or non-CV disorders were also excluded, as well as studies that did not report the specified outcomes. Ongoing studies and studies with a follow-up period of less than 12 months were not included. This search strategy ensured the selection of high-quality studies, allowing for a comprehensive analysis of the impact of statin therapy on CV outcomes.

Outcome Variables

The primary clinical outcomes were all-cause mortality, CV mortality, MACE, and CV rehospitalization. Outcome definitions followed those provided by each study. We assessed the longest available follow-up based on per-protocol criteria.

Data Extraction

We independently extracted the first author's name, publication year, place of origin, sample size, and follow-up period. Information on participants' baseline demographics (age, gender, ethnicity), CV risk factors (hypertension, diabetes, hyperlipidemia), CVDs (heart failure, coronary artery disease), chronic kidney disease (CKD), and diabetes mellitus was collected. Details on the intervention, including statin type, dosage, duration, intensity (low, moderate, or high), and comparator groups (placebo, non-statin medication, or no intervention), were also extracted.

The outcome measures, such as all-cause mortality, MACE (heart attack, strokes, coronary revascularization, and other CV events), and CV hospitalization, along with any secondary outcomes from the studies, were meticulously recorded. Two independent reviewers extracted the data to minimize errors, and any discrepancies were resolved through discussion or by involving a third reviewer. This systematic approach to data extraction ensured that all key information was gathered for a thorough and accurate systematic review and meta-analysis.

Risk-of-Bias Assessment

The risk of bias in RCTs was assessed by the same investigators for each study, independently using the Cochrane risk-of-bias tool [9]. The evaluated factors included random sequence generation (selection bias), allocation sequence concealment (selection bias), blinding of participants and personnel (performance bias), blinding of outcome assessment (detection bias), incomplete outcome data (attrition bias), selective outcome reporting (reporting bias), and other potential sources of bias. The risk of bias for each study was categorized as "low," "high," or "unclear."

For cohort studies, the Newcastle-Ottawa Scale (NOS) was used to evaluate risk of bias. This assessment focused on three key domains: (1) selection, which included the representativeness of the exposed cohort, selection of the non-exposed cohort, ascertainment of exposure, and confirmation that the outcome of interest was not present at the start of the study; (2) comparability of the exposed and non-exposed groups; and (3) exposure, including the assessment of outcomes, adequacy of follow-up duration for outcomes to occur, and completeness of cohort follow-up. Each study's risk of bias was rated as "good," "fair," or "poor."

For observational studies, we evaluated how each study addressed potential confounding factors. This included reporting of adjustment methods such as multivariable regression models and, in some cases, propensity score matching. However, as these methods varied across studies, residual confounding may still be present and should be taken into account when interpreting the results.

Statistical Analysis

A two-tailed p < 0.05 was considered significant [10]. Meta-analyses were performed with random-effects models, as we expected heterogeneity of effects among studies. The generic inverse variance method was used to determine the risk ratios for various outcomes. Heterogeneity between studies was assessed using the I² statistic. As a guide, I2 < 25% indicates low, 25-50% moderate, and >50% high heterogeneity [11]. Meta-analyses were conducted using RevMan 5.1 (The Cochrane Collaboration, Copenhagen, Denmark).

While the small number of included studies limited the feasibility of formal subgroup analyses, we considered subgroup comparisons based on statin type (lipophilic vs. unspecified), study design (RCT vs. observational), and population characteristics (e.g., heart failure patients). Sensitivity analysis was conducted by evaluating the impact of excluding any single study from the meta-analysis, although due to the consistent direction of effect, the results were robust. Further subgroup-level meta-analyses were not performed due to insufficient disaggregated data across studies.

Dataset and Visualization

All data included in this review and meta-analysis were extracted manually from eligible studies following predefined inclusion criteria. The pooled data were analyzed using Review Manager (RevMan) version 5.1, a tool developed by the Cochrane Collaboration for systematic reviews and meta-analyses. All forest plots and figures in this manuscript were generated using RevMan.

No new datasets were created, and all data supporting the findings of this study are available from the corresponding published studies, as cited. References to each included study are provided in the main reference list, and summary data are available upon reasonable request from the corresponding author.

Study Registration and Ethical Considerations

The study protocol was not registered in PROSPERO or any other systematic review registry. While this limits transparency in protocol adherence, the study was conducted in accordance with PRISMA guidelines and adhered to established methodological standards.

As the study involved analysis of previously published data and did not involve direct contact with human subjects, institutional review board (IRB) approval and informed consent were not required. Ethical principles of data use and reporting integrity were strictly followed throughout.

Results

Search Results and Trial Flow

**Figure 1:**
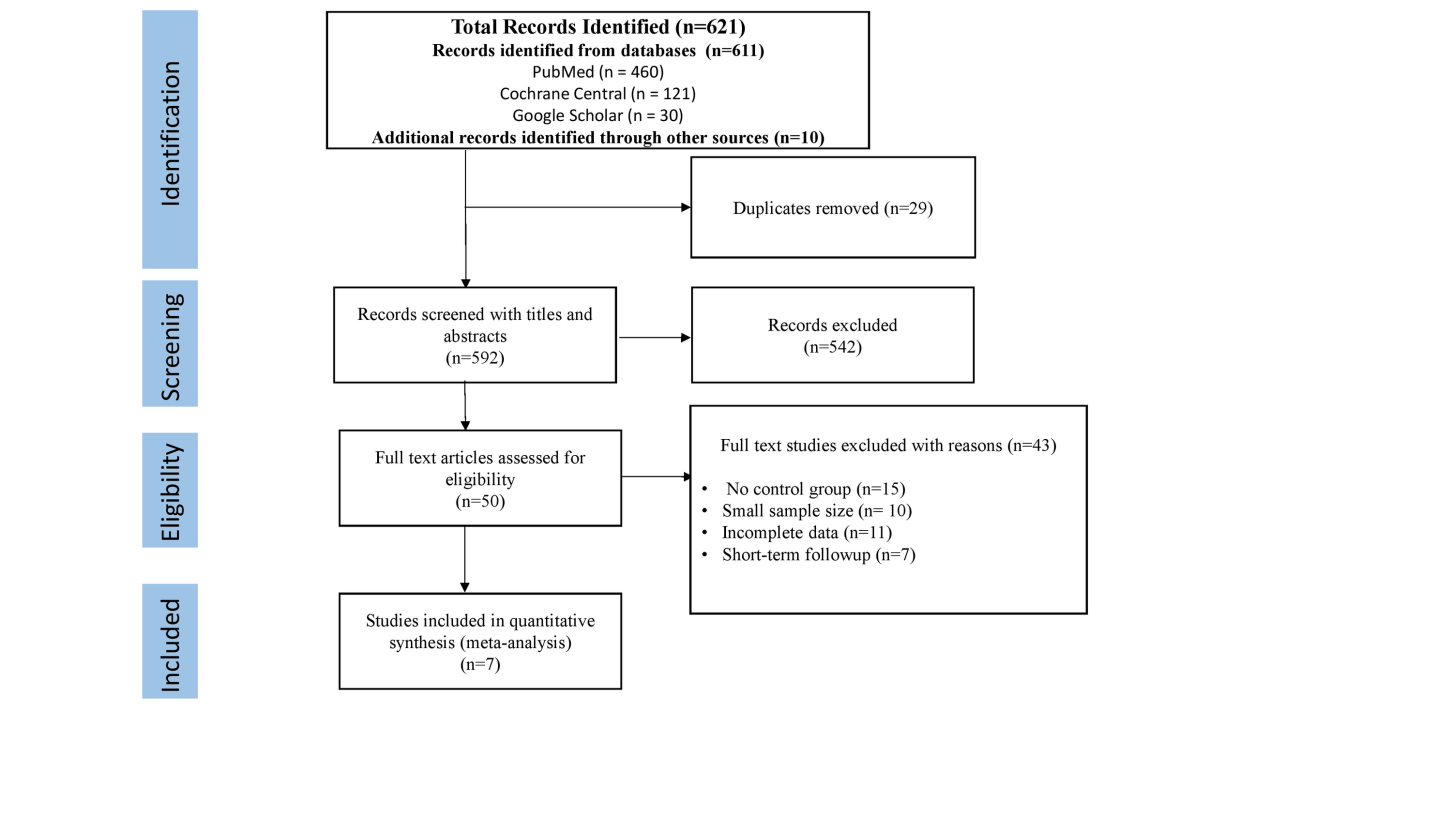
PRISMA flow diagram depicting the study selection process A total of 621 records were identified, including 460 from PubMed, 121 from Cochrane Central Registry, and 30 from Google Scholar. An additional 10 records were identified through manual searches of bibliographies and other sources. After removing 29 duplicates, 592 records were screened by title and abstract. Of these, 542 were excluded, and 50 full-text articles were assessed for eligibility. Ultimately, seven studies were included in the meta-analysis. Reasons for exclusion at the full-text stage are detailed in the diagram. Image credits: Priyadeep Kaur

This systematic review initially identified 621 records, with 611 obtained through database searches and 10 from other sources (Figure 1). After removing 29 duplicates, 592 records remained for the title and abstract screening. At this stage, 542 records were excluded, leaving 50 for full-text assessment. Ultimately, seven studies met the inclusion criteria [12-18], consisting of three RCTs [12-14], two prospective [15,16], and two retrospective cohort studies [17,18].

Characteristics of the Included Studies

Seven studies with a total of 506,813 patients (118,491 treated with statins and 388,322 without statins), with a mean follow-up of 3.7 years, were ultimately included in the meta-analysis (Table 1). The mean age of patients was 69.4±8.6 years, 96.9% were male, 70.4% had arterial hypertension, 34.2% had diabetes, and 4.7% had CKD (Table 2). Studies by Castellano et al. (2022) [12], Hamada et al. (2023) [13], and Selvaraj et al. (2022) [14] evaluated lipophilic statins, whereas the remaining studies did not specify the type of statin used.

**Table 1:** Main characteristics of the studies included in the meta-analysis RCT: randomized controlled trial; MACE: major adverse cardiovascular events; CKD: chronic kidney disease; BMI: body mass index

Study/Year	Country	Study Design	Inclusion Criteria	Study Comparison	Types of Statins	Primary Endpoints	Follow-up	Key Study Limitations
Castellano et al., 2022 [12]	European countries	RCT	Myocardial infarction	Statins: Control	Atorvastatin	All-cause mortality, cardiovascular mortality, MACE (myocardial infarction, ischemic stroke, urgent revascularization)	3 years	Statin dose and intensity not stratified; CKD status not reported
Hamada et al., 2023 [13]	Japan	RCT	Chronic hemodialysis with dyslipidemia	Statins: Control	Pitavastatin	All-cause mortality, cardiovascular mortality, MACE (nonfatal myocardial infarction, nonfatal stroke, coronary revascularization), cardiovascular rehospitalization	3 years	Small sample size; limited generalizability to non-dialysis patients
Selvaraj et al., 2022 [14]	United States of America	RCT	Patients with/without heart failure having a history of atrial fibrillation	Statins: Control	Icosapent ethyl	Cardiovascular mortality, MACE (nonfatal myocardial infarction, nonfatal stroke, coronary revascularization), cardiovascular rehospitalization	2 years	Statin not standard; short follow-up; statin type not widely used
Marume et al., 2019 [15]	Japan	Prospective cohort study	Acute heart failure without coronary artery disease	Statins: Control	Not specified	All-cause mortality, cardiovascular rehospitalization	2 years	Very small sample size; statin type and dosage not reported
Nochioka et al., 2015 [16]	Japan	Prospective cohort study	Heart failure with preserved ejection fraction	Statins: Control	Not specified	All-cause mortality, cardiovascular mortality, cardiovascular rehospitalization	3 years and 4 months	Observational design; statin details (type/intensity) not reported
Orkaby et al., 2020 [17]	United States of America	Retrospective cohort study	75 years and older veterans without atherosclerotic cardiovascular disease	Statins: Control	Any new statin prescription	All-cause mortality, cardiovascular mortality, MACE	6 years and 8 months	Male-dominant population; residual confounding likely
Jung et al., 2021 [18]	Korea	Retrospective cohort study	Predicted cardiovascular disease risk	Statins: Control	Not specified	All-cause mortality, MACE	6 years	Missing BMI and CKD data; lack of adjustment for lifestyle factors

**Table 2:** Main characteristics of the patients enrolled among the studies included in the meta-analysis S: statin Group; C: control group; NR: not reported; BMI: body mass index; HTN: hypertension; DM: diabetes mellitus; CKD: chronic kidney disease

Study/Year	Arms	No.	Age (Years)	BMI	Male%	HTN%	DM%	CKD%
Castellano et al., 2022 [12]	S	1237	75.8±6.7	27.4±4.4	69%	77.0%	42%	NR
	C	1229	76.1±6.5	27.5±4.3	69%	78.8%	43.2%	NR
Hamada et al., 2023 [13]	S	426	61.0±10.8	NR	60%	88.2%	NR	NR
	C	422	59.7±11.0	NR	61.4%	93.3%	NR	NR
Selvaraj et al., 2022 [14]	S	703	63.0	31.0	69.3%	93.7%	53.9%	27%
	C	743	63.0	31.0	69.3%	85.1%	58.7%	21.2%
Marume et al., 2019 [15]	S	56	78.0±9	24.6±4.4	19%	89%	36%	NR
	C	56	76.0±10	24.6±5.3	22%	86%	36%	NR
Nochioka et al., 2015 [16]	S	626	69.4±11.2	24.2±3.6	65.5%	82.7%	28.9%	NR
	C	626	70.0±11.9	24.2±4.1	67%	80.7%	26.0%	NR
Orkaby et al., 2020 [17]	S	57178	81.1±4.1	27.4±3.6	97.3%	77.8%	23.4%	1.9%
	C	326981	81.5±1.5	27.4±1.6	97.3%	77.8%	23.4%	1.9%
Jung et al., 2021 [18]	S	58265	59.2 ± 9.0	NR	45.5%	69.2%	28.7%	NR
	C	58265	58.2±9.1	NR	46.2%	39.7%	13.7%	NR

Clinical Outcomes

**Figure 2:**
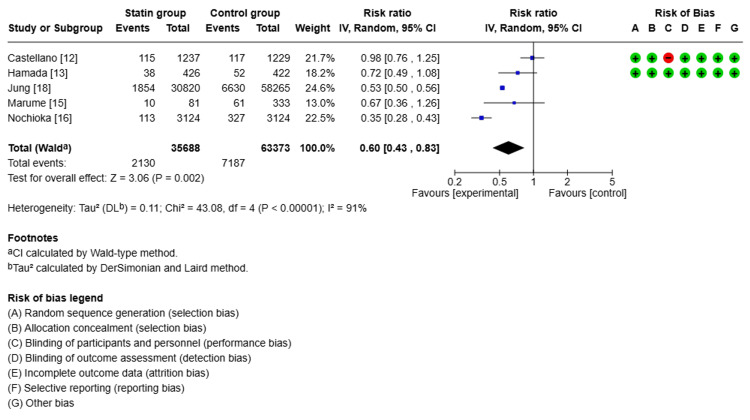
Comparison of all-cause mortality in the statin group versus the non-statin group

**Figure 3:**
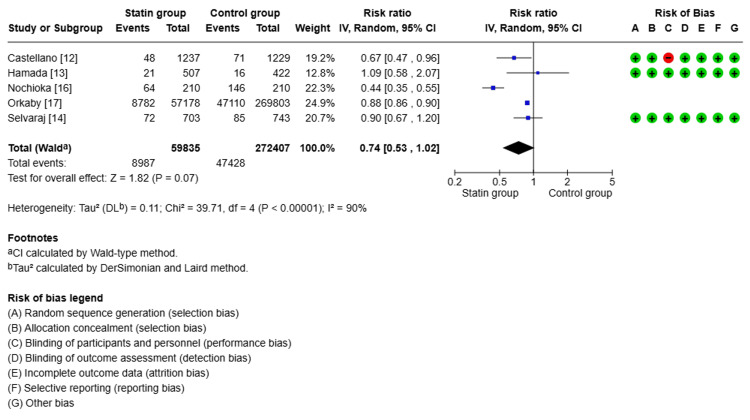
Comparison of cardiovascular mortality in the statin group versus the non-statin group

**Figure 4:**
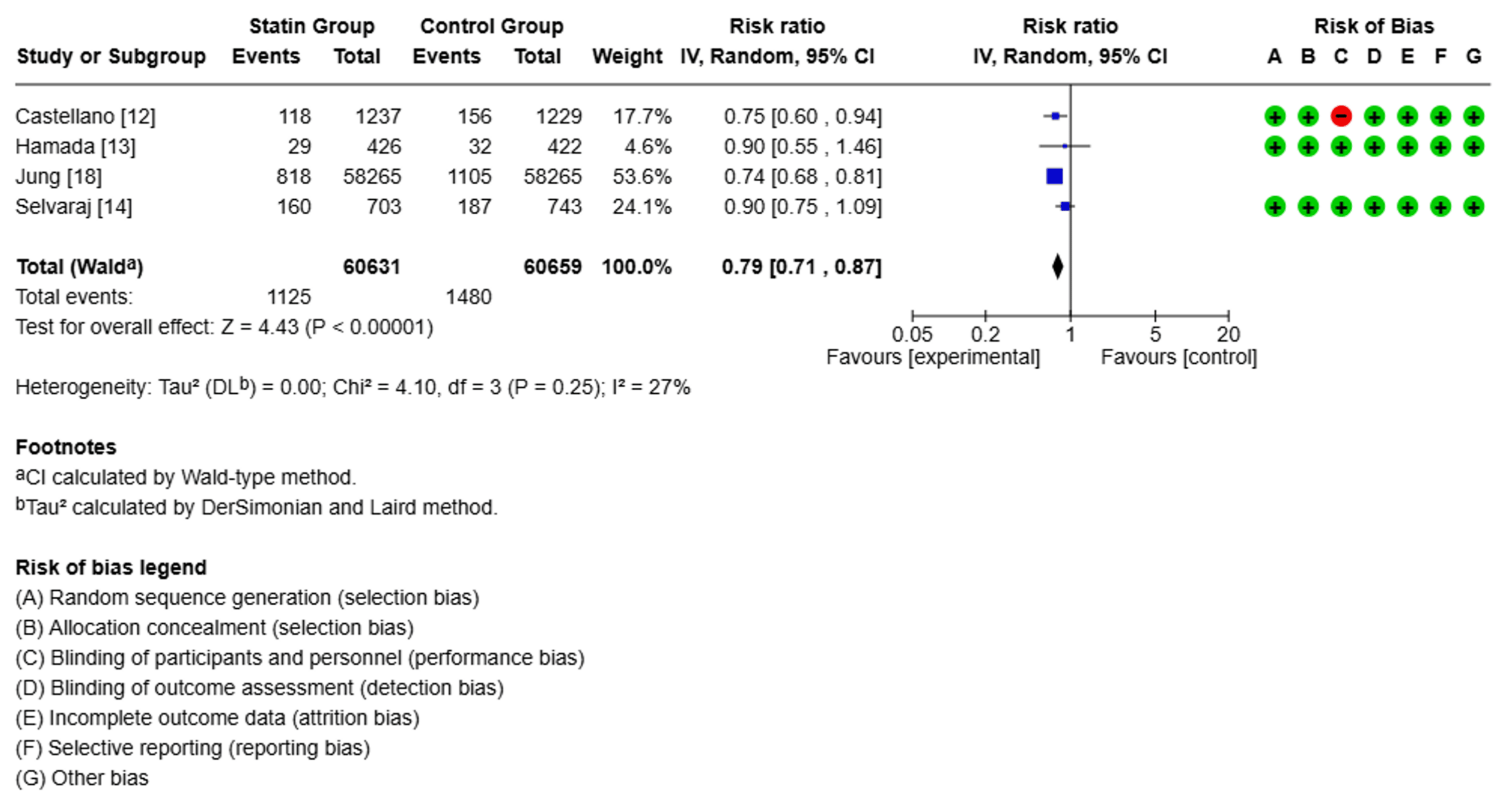
Comparison of major cardiovascular events (MACE) in the statin group versus the non-statin group

**Figure 5:**
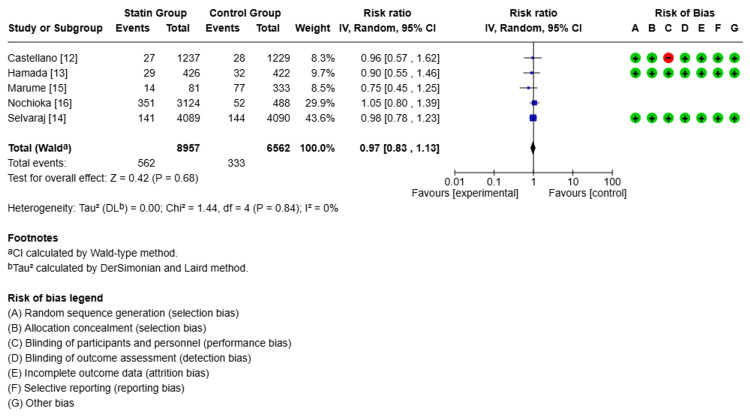
Comparison of cardiovascular hospitalization in the statin group versus the non-statin group

Follow-up ranged from two years to six years and eight months, with a mean of three years and seven months. Compared with non-statin users, statin users showed a 40% lower risk of all-cause mortality (relative risk (RR) 0.60, 95% confidence interval (CI): 0.43-0.83, p<0.00001, I²=91%, Figure 2). For CV mortality, the pooled estimate suggested a 26% lower risk with statin use (RR 0.74, 95% CI: 0.53-1.02, p<0.00001, I²=90%, Figure 3). However, the CI included 1.0, indicating that this result was not statistically significant, despite the low p-value. Statin therapy was also associated with a 21% lower risk of MACE (RR 0.79, 95% CI: 0.71-0.87, p<0.00001, I²=0%, Figure 4). Statin use did not show a statistically significant effect on reducing CV hospitalization (RR 0.97, 95% CI: 0.83-1.13, p<0.00001, I²=0%, Figure 5).

Due to the high heterogeneity observed in all-cause and CV mortality (I²=91% and 90%, respectively), a sensitivity analysis was conducted by sequentially excluding each study to assess the influence of individual studies on the overall effect size. The direction of effect remained consistent, suggesting the robustness of the pooled estimate. However, heterogeneity remained high.

Subgroup comparisons were considered but limited by the small number of included studies. Nonetheless, a basic stratification by statin type (lipophilic vs. unspecified) and study design (RCT vs. observational) suggested that differences in drug pharmacology and methodology might have contributed to heterogeneity. Further exploration of these factors is warranted in larger meta-analyses.

Risk-of-Bias Assessment

The three included RCTs had a low risk of bias, and the observational studies showed good quality (Tables 3, 4).

**Table 3:** Risk of bias in the included RCTs

Study	Random Sequence Generation	Allocation Concealment	Blinding of Participants and Personnel	Blinding of Outcome Assessment	Incomplete Outcome Data	Selective Reporting	Other Bias	Overall
Castellano et al., 2022 [12]	Low	Low	High	Low	Low	Low	Low	Low
Hamada et al., 2023 [13]	Low	Low	Low	Low	Low	Low	Low	Low
Selvaraj et al., 2022 [14]	Low	Low	Low	Low	Low	Low	Low	Low

**Table 4:** Quality assessment of the included prospective and retrospective studies

Study	Selection				Comparability	Outcome of Interest			Overall Quality of Study
	Representativeness of the exposed cohort	Selection of the non-exposed cohort	Ascertainment of exposure	Outcome present at start of study	Comparability of cohorts	Assessment of outcome	Length of follow-up	Adequacy of follow-up	
Marume et al., 2019 [15]	Good	Good	Poor	Good	Good	Good	Good	Good	Good
Nochioka et al., 2015 [16]	Good	Good	Good	Good	Good	Good	Good	Good	Good
Orkaby et al., 2020 [17]	Good	Good	Good	Good	Good	Good	Good	Good	Good
Jung et al., 2021 [18]	Good	Good	Good	Good	Good	Good	Good	Good	Good

Discussion

This systematic review evaluated the impact of statin therapy compared to non-statin therapy on clinical outcomes in patients with CV risk. Statin therapy was associated with significant reductions in all-cause mortality, CV mortality, and MACE. However, it did not demonstrate a statistically significant reduction in CV hospitalizations. These findings highlight the established benefits of statins in improving survival and mitigating CV risks while also revealing certain areas where their impact is less clear.

The observed reductions in mortality and MACE are underpinned by well-documented biological mechanisms. Statins act by inhibiting HMG-CoA reductase, the key enzyme involved in cholesterol synthesis, resulting in decreased LDL cholesterol levels. This reduction slows the progression of atherosclerosis, which is the primary pathological process underlying most CV events, including myocardial infarction and stroke [19]. By stabilizing atherosclerotic plaques, reducing systemic inflammation, and enhancing endothelial function, statins lower the risk of plaque rupture and thrombosis, thereby reducing the incidence of CV events. These mechanisms contribute significantly to the observed decrease in mortality and MACE [20,21].

In addition to their lipid-lowering properties, statins exhibit a range of pleiotropic effects that may independently contribute to CV risk reduction. These include anti-inflammatory, antioxidative, antithrombotic, and plaque-stabilizing actions. Statins reduce systemic inflammation by decreasing levels of C-reactive protein (CRP), an independent marker of CV risk [22]. For instance, the JUPITER trial showed that rosuvastatin significantly reduced CRP levels, which was associated with a lower incidence of vascular events, even in individuals with normal LDL levels [23]. Additionally, statins improve endothelial function by upregulating endothelial nitric oxide synthase (eNOS), leading to better vasodilation and reduced vascular stiffness. Their antioxidative properties counteract oxidative stress in vascular walls by inhibiting the production of reactive oxygen species. Furthermore, statins enhance plaque stability by decreasing macrophage infiltration and increasing the thickness of the fibrous cap, thereby reducing the risk of plaque rupture and subsequent thrombosis [21]. These effects collectively help explain the reduction in MACE, even in the absence of dramatic LDL changes, and underscore the broad utility of statins in CV prevention.

While statins are generally well tolerated, their adverse effects should not be overlooked. Common side effects include myalgias, elevated liver enzymes, and, less frequently, new-onset diabetes mellitus. Rare but serious complications like rhabdomyolysis can also occur. These adverse effects may influence adherence and treatment discontinuation, potentially affecting clinical outcomes. However, the included studies reported limited or no data on statin-related side effects, which restricted our ability to systematically evaluate their safety profile.

Despite their well-established benefits, statins did not show a statistically significant reduction in CV hospitalizations in this meta-analysis. Several multifactorial reasons may account for this finding. Hospitalization rates are influenced not only by the occurrence of CV events but also by a variety of other factors, including the presence of comorbidities, the severity of illness, patient adherence to treatment, and accessibility to healthcare services. For instance, patients with well-controlled LDL levels on statin therapy may still require hospitalization for non-CV conditions or other complications, such as heart failure exacerbations. Furthermore, variability across healthcare systems and differences in study designs may have introduced heterogeneity that impacted this outcome.

The results of this study align with findings from other recent meta-analyses that underscore the benefits of statin therapy. For example, Nowak et al. reported a significant 30% reduction in all-cause mortality with statin use, consistent across both CVD and non-CVD cohorts. However, their analysis also noted substantial heterogeneity in the outcomes, a finding similar to that in our study [24]. Andersson et al. demonstrated that statin therapy was associated with significant reductions in all-cause mortality, CV mortality, and MI risk, particularly during the initial four years of follow-up. These benefits were even more pronounced in women, where reductions in mortality and MI risk were associated with favorable numbers needed to treat [25].

Another systematic review and meta-analysis involving Asian patients with coronary artery stenosis revealed that statin therapy significantly reduced adverse CV events. Sensitivity analyses confirmed these findings, although the authors noted that trial sequential analysis indicated the need for larger sample sizes to validate the results further [26]. Anderson et al. examined the role of statins in a cohort of patients with HFrEF. Despite a higher prevalence of baseline ASCVD (atherosclerotic CV disease) risk factors among statin users, the study found a significant association between statin use and a reduced risk of MACE, even after adjusting for baseline differences [27].

While this meta-analysis highlights the benefits of statins, several limitations must be acknowledged. Inter-study variability was evident across several parameters, including population characteristics, baseline CV risk, follow-up duration, and types and intensities of statin therapy. This variability likely contributed to the high heterogeneity observed in some outcomes, particularly all-cause and CV mortality. For example, some studies used specific types of statins, such as lipophilic statins, while others did not specify the type used, potentially contributing to the observed differences in outcomes.

Additionally, the pooled population was predominantly male (96.9%), reflecting recruitment patterns in CV trials. This gender imbalance limits the generalizability of findings to female patients, who may exhibit different responses to statins in terms of efficacy and side-effect profile. Future studies should aim to include more balanced gender representation to support broader applicability.

Furthermore, due to inconsistent reporting across studies, we were unable to perform detailed subgroup analyses based on statin type (e.g., lipophilic vs. hydrophilic), which could have influenced the observed outcomes and heterogeneity. Information on patient characteristics, such as adherence to statin therapy, specific statin dosages, or baseline LDL levels, was frequently unavailable. Moreover, several key baseline characteristics, such as body mass index (BMI) and the presence of CKD, were not consistently reported across the included studies. This incomplete data limited our ability to fully adjust or stratify for these important covariates, potentially contributing to unexplained heterogeneity and restricting deeper subgroup analyses. The included observational studies, while of high quality, lacked the rigorous control over confounding factors that is typically achieved in RCTs. This inherent limitation may have influenced the pooled outcomes and contributed to the observed heterogeneity.

Despite these limitations, this study provides valuable insights into the role of statins in reducing mortality and MACE among patients with CV risk. The findings align with well-established evidence supporting the benefits of statins as a cornerstone of therapy for primary and secondary prevention of CVDs. The lack of a significant reduction in CV hospitalizations highlights the need for further research to better understand the multifactorial factors influencing this outcome.

This systematic review and meta-analysis underscore the efficacy of statins in reducing all-cause mortality, CV mortality, and MACE among patients with CV risk. The findings reinforce the importance of statins in contemporary CV management. Future studies should aim to address the gaps identified in this analysis, such as the impact of different statin types and dosages, patient adherence, and healthcare system variability, to provide a more comprehensive understanding of the broader implications of statin therapy.

## Conclusions

This meta-analysis highlights the substantial benefits of statin therapy in reducing all-cause mortality and MACE, reinforcing its essential role in managing CVD. Statins, associated with a 40% reduction in all-cause mortality and a 25% decrease in heart attacks, strokes, and other major CV events, have become standard in both primary and secondary prevention. Although a trend toward reduced CV mortality was observed, it did not reach statistical significance, indicating the need for further large-scale studies to clarify this effect. Notably, statins did not reduce CV hospitalizations, suggesting that additional interventions may be necessary to lower admission rates. Clinically, statins should be prescribed broadly to high-risk CVD patients, with individualized approaches based on specific patient characteristics and risk profiles. This review emphasizes the importance of long-term adherence, ongoing side-effect monitoring, and keeping patients informed to optimize outcomes.

Future research should focus on the long-term effects of statins, evaluate their impact across diverse subpopulations, and explore the full scope of their benefits. Such studies will advance treatment options and solidify statin therapy as a frontline tool in the fight against the global burden of CVD. The widespread use of statins by clinicians and health organizations remains a priority to improve patient outcomes and reduce CV mortality.

## References

[1] Di Cesare M, Perel P, Taylor S (2024). The heart of the world. Glob Heart.

[2] Mensah GA, Wei GS, Sorlie PD (2017). Decline in cardiovascular mortality: possible causes and implications. Circ Res.

[3] Bays HE, Taub PR, Epstein E (2021). Ten things to know about ten cardiovascular disease risk factors. Am J Prev Cardiol.

[4] Somers T, Siddiqi S, Morshuis WJ, Russel FG, Schirris TJ (2023). Statins and cardiomyocyte metabolism, friend or foe?. J Cardiovasc Dev Dis.

[5] Taylor F, Huffman MD, Macedo AF (2013). Statins for the primary prevention of cardiovascular disease. Cochrane Database Syst Rev.

[6] Chou R, Cantor A, Dana T, Wagner J, Ahmed AY, Fu R, Ferencik M (2022). Statin use for the primary prevention of cardiovascular disease in adults: updated evidence report and systematic review for the US Preventive Services Task Force. JAMA.

[7] Yourman LC, Cenzer IS, Boscardin WJ (2021). Evaluation of time to benefit of statins for the primary prevention of cardiovascular events in adults aged 50 to 75 years: a meta-analysis. JAMA Intern Med.

[8] Moher D, Liberati A, Tetzlaff J, Altman DG (2009). Preferred reporting items for systematic reviews and meta-analyses: the PRISMA statement. PLoS Med.

[9] Cumpston M, Li T, Page MJ, Chandler J, Welch VA, Higgins JP, Thomas J (2019). Updated guidance for trusted systematic reviews: a new edition of the Cochrane Handbook for Systematic Reviews of Interventions. Cochrane Database Syst Rev.

[10] Kwak S (2023). Are only p-values less than 0.05 significant? A p-value greater than 0.05 is also significant!. J Lipid Atheroscler.

[11] Higgins JP, Thompson SG, Deeks JJ, Altman DG (2003). Measuring inconsistency in meta-analyses. BMJ.

[12] Castellano JM, Pocock SJ, Bhatt DL (2022). Polypill strategy in secondary cardiovascular prevention. N Engl J Med.

[13] Hamada C, Okuda M, Tomino Y (2023). Pitavastatin compared with differential intervention trial by standard therapy on cardiovascular events in patients with dyslipidemia on chronic hemodialysis (DIALYSIS): a randomized controlled trial. Blood Purif.

[14] Selvaraj S, Bhatt DL, Steg PG (2022). Impact of icosapent ethyl on cardiovascular risk reduction in patients with heart failure in REDUCE-IT. J Am Heart Assoc.

[15] Marume K, Takashio S, Nagai T, Tsujita K, Saito Y, Yoshikawa T, Anzai T (2019). Effect of statins on mortality in heart failure with preserved ejection fraction without coronary artery disease ― report from the JASPER study. Circ J.

[16] Nochioka K, Sakata Y, Miyata S (2015). Prognostic impact of statin use in patients with heart failure and preserved ejection fraction. Circ J.

[17] Orkaby AR, Driver JA, Ho YL (2020). Association of statin use with all-cause and cardiovascular mortality in US veterans 75 years and older. JAMA.

[18] Jung HH (2021). Statin use and outcome risks according to predicted CVD risk in Korea: a retrospective cohort study. PLoS One.

[19] Khatiwada N, Hong Z (2024). Potential benefits and risks associated with the use of statins. Pharmaceutics.

[20] Zhou Q, Liao JK (2009). Statins and cardiovascular diseases: from cholesterol lowering to pleiotropy. Curr Pharm Des.

[21] Takata K, Imaizumi S, Zhang B, Miura S, Saku K (2016). Stabilization of high-risk plaques. Cardiovasc Diagn Ther.

[22] Morofuji Y, Nakagawa S, Ujifuku K, Fujimoto T, Otsuka K, Niwa M, Tsutsumi K (2022). Beyond lipid-lowering: effects of statins on cardiovascular and cerebrovascular diseases and cancer. Pharmaceuticals (Basel).

[23] Ridker PM, Danielson E, Fonseca FA (2008). Rosuvastatin to prevent vascular events in men and women with elevated C-reactive protein. N Engl J Med.

[24] Nowak MM, Niemczyk M, Florczyk M, Kurzyna M, Pączek L (2022). Effect of statins on all-cause mortality in adults: a systematic review and meta-analysis of propensity score-matched studies. J Clin Med.

[25] Andersson T, Nåtman J, Mourtzinis G, Bager JE, Bengtsson Boström K, Franzén S, Hjerpe P (2023). The effect of statins on mortality and cardiovascular disease in primary care hypertensive patients without other cardiovascular disease or diabetes. Eur J Prev Cardiol.

[26] Aslani S, Razi B, Imani D, Mohammadi K, Jamialahmadi T, Reiner Ž, Sahebkar A (2023). Effect of statins on the blood lipid profile in patients with different cardiovascular diseases: a systematic review with meta-analysis of randomized clinical trials. Curr Med Chem.

[27] Anderson JL, May HT, Le VT (2023). Impact of statin therapy in heart failure patients: results of a large real-world experience. JACC Adv.

