# Correction: Effect of Statin Therapy on Clinical Outcomes in Patients With Cardiovascular Risks: A Systematic Review and Meta-Analysis

**DOI:** 10.7759/cureus.c461

**Published:** 2026-07-10

**Authors:** Kiyan Ghani Khan, Priyadeep Kaur, Mehak Bhagat, Huda K Klair, Maryam Bakhtawar, Jorge Manuel Aldea Saldaña, Hasiya M Bello, Hussein Attia Hussein Mahmoud, Mahesh Babu, Tanvi Mahajan, Usman Khan, Manju Rai

**Affiliations:** 1 Internal Medicine, Baqai Medical University, Karachi, PAK; 2 Internal Medicine, Punjab Institute of Medical Sciences, Jalandhar, IND; 3 Internal Medicine, Government Medical College, Amritsar, Amritsar, IND; 4 Internal Medicine, Fatima Memorial Hospital (FMH) College of Medicine and Dentistry, Lahore, PAK; 5 Internal Medicine, Beihua Medical University, Jilin City, CHN; 6 Cardiology, Universidad Peruana de Ciencias Aplicadas, Lima, PER; 7 Emergency Medicine, Buraydah Central Hospital, Buraydah, SAU; 8 Diagnostic Radiology, Heliopolis Hospital, Cairo, EGY; 9 Internal Medicine, Dr. PSI Medical College, Andhra Pradesh, IND; 10 Internal Medicine, Maharishi Markandeshwar Medical College and Hospital, Dinanagar, IND; 11 General Practice, Akhtar Saeed Medical and Dental College, Lahore, PAK; 12 Biotechnology, Shri Venkateshwara University, Gajraula, IND

This article has been corrected to fix two instances of relative risk (RR) that were incorrectly entered: one each in the Abstract and Results section. Statin therapy was associated with a 21% lower risk of MACE (RR 0.79, 95% CI: 0.71–0.87).

